# Dabrafenib in metastatic melanoma: a monocentric ‘real life’ experience

**DOI:** 10.3332/ecancer.2016.624

**Published:** 2016-03-03

**Authors:** E Cocorocchio, S Gandini, S Alfieri, A Battaglia, E Pennacchioli, G Tosti, G Spadola, M Barberis, M Di Leo, C Riviello, L Pala, A Intelisano, C Martinoli, PF Ferrucci

**Affiliations:** 1 Medical Oncology of Melanoma and Sarcoma Division, Istituto Europeo di Oncologia, via Ripamonti 435, Milan 2014, Italy; 2 Biostatistics Division, Istituto Europeo di Oncologia, via Ripamonti 435, Milan 2014, Italy; 3 Sarcoma Unit, Istituto Europeo di Oncologia, via Ripamonti 435, Milan 2014, Italy; 4 Dermatoncological Surgery Division, Istituto Europeo di Oncologia, via Ripamonti 435, Milan 2014, Italy; 5 Pathology Division, Istituto Europeo di Oncologia, via Ripamonti 435, Milan 2014, Italy

**Keywords:** metastatic melanoma, Dabrafenib, BRAF V600 mutation, target therapy

## Abstract

Dabrafenib is a potent BRAF-kinase inhibitor. Its activity was evaluated on 40 consecutive metastatic melanoma patients (pts) harboring the V600BRAF mutations. Dabrafenib was administered orally at the dosage of 150 mg b.i.d. daily. ORR was 82%, with 7% CR, 62% PR, 13% SD and 18% PD. The median PFS and OS were seven and 17 months, respectively (median follow-up: 8.5 months). Increased risk of progression was found in pts with elevated LDH, ECOG PS >1 and more than two metastatic sites. Grade 3–4 adverse events were recorded in 4 pts. In this retrospective analysis, Dabrafenib confirmed its role as the standard clinical option in metastatic melanoma pts.

## Introduction

Metastatic melanoma (MM) is an incurable disease, which standard chemotherapies failed to obtain encouraging results with [1]. After detecting crucial mechanisms that regulate the survival and growth of tumor cells and their interaction with their own microenvironment and the patient’s immune system, new effective drugs were developed and introduced into clinical practice.

BRAF is a critical kinase in the mitogen-activated protein kinase (MAPK) pathway that influences the transcription and translation of a number of proteins that regulate cell proliferation, differentiation and migration [2].

Melanoma progression can depend on BRAF mutations, which are able to induce and maintain the activation of the MAPK pathway in the absence of growth factor signals. The most frequent mutations are the substitution of valine in position 600 with glutamate or lysine (V600E; V600K) [3]. On the basis of the evidence that these oncogenic BRAF mutations are detectable in about 50% of patients (pts) with cutaneous melanoma [4], research was focused on the inhibition of this pathway in order to reverse uncontrolled growth of melanoma cells. Vemurafenib was the first BRAF V600 inhibitor available in the clinical setting. In the phase III clinical trial this treatment was compared with Dacarbazine (DTIC). Clinical results were recently updated, and confirmed the benefit for the Vemurafenib arm, in terms of median progression free survival (PFS) (6.9 versus 1.6 months, respectively, with Hazard Ratio (HR) of 0.38 (95% CI: 0.32–0,46; p < 0.0001)), and median overall survival (OS) (13.6 versus 9.7 months, respectively, with HR 0.70 (95% CI: 0.57–0.87; p = 0.0008)) [5]. Furthermore, Vemurafenib demonstrated activity also in pts with central nervous system (CNS) metastases [6]. Dabrafenib (GSK2118436) is another potent ATP-competitive inhibitor of BRAF kinase that demonstrated activity in patients with V600E and V600K BRAF-mutant melanoma. The phase III trial comparing Dabrafenib with DTIC demonstrated a median PFS of 5.1 and 2.7 months, for the two drugs, respectively, with HR of 0.30 (95% CI: 0.18-0.51; p < 0.0001) [7]. Also these results were recently updated [8], and the improvement was confirmed in terms of median PFS (6.9 and 2.7 months, respectively), with a HR of 0.37 (95% CI: 0.23-0.57; p < 0.0001) in favor of Dabrafenib, while median OS was 18.2 versus 15.6 months, respectively [8]. Dabrafenib is also active in the presence of CNS metastases, either progressive or not, after radiotherapy or surgery: the intracranial disease control was obtained in more than 80% of BRAFV600E mutated pts [9].

Although the efficacy of these two inhibitors seems to be similar, their toxicity profile looks different: Vemurafenib induces rush, photosensitivity, arthralgia, fatigue, alopecia, nausea and diarrhea, whereas Dabrafenib induces palmar-plantar erythrodysaesthesia (PPE), palmar-plantar hyperkeratosis (PPH), fever, fatigue, arthralgia and headache. The incidence of appearance and progression of proliferative skin lesions such as keratoacanthomas and cutaneous squamous cell carcinomas (cSCC) seems to be higher with Vemurafenib [5, 7]. The observation that one of the resistance mechanisms to BRAF inhibitors and the arising of proliferative skin lesion were due to a paradoxical reactivation of the MAPK pathway, justified the rationale of associating BRAF and MEK inhibitors [10]. Three randomized clinical trials [11–13] comparing BRAF inhibitor monotherapy with BRAF/MEK inhibitors association were published, demonstrating a better outcome for combinations. Furthermore, all these trials demonstrated that BRAF/MEK inhibitors combination seems to modify the toxicity profile of BRAF inhibitors alone, by reducing the incidence of cSCC and other cutaneous proliferative disorders [11, 14]. Thus, the combination of BRAF/MEK inhibitors is now considered to be the standard clinical option in metastatic BRAF V600 mutated pts.

On this basis, for pts with metastatic melanoma harboring BRAF V600 mutation, a compassionate use program with Dabrafenib first and Dabrafenib and Trametinib later were started.

The aim of this retrospective analysis is to to investigate if real life data support the findings of clinical studies on the effect of Dabrafenib as monotherapy.

## Patients and methods

We analyzed the data of 40 consecutive pts with MM who were treated with Dabrafenib at the Istituto Europeo di Oncologia. The pts included were ≥18 years of age, had confirmed BRAF V600 mutation, an Eastern Cooperative Oncology Group (ECOG) performance status (PS) of 0–3, were able to swallow and retain oral medications, and were free of medical conditions or clinical laboratory findings which would imply a risk for them of an adverse outcome. Mutation analysis on exons 11 and 15 of BRAF gene was performed by bi-directional Sanger sequencing. Mutations were confirmed by pyrosequencing using the ‘BRAF status kit’ (Diatech Pharmacogenetics, Jesi, Italy) running on PyroMark Q96 ID system (Qiagen, Hiden, Germany) according to the manufacturer’s instructions. Patients with brain metastases, not requiring or ineligible for immediate local treatment, were included. Pts had to agree to use adequate contraception during therapy. Exclusion criteria were: pregnancy, prior therapy with BRAF inhibitors, and known or suspected hypersensitivity to any component of Dabrafenib. Before therapy, all pts underwent total body CT-scans, blood cell count and chemistry, including total proteins, albumin, potassium, sodium, calcium, glucose, urea, creatinine, total serum bilirubin, aspartate transaminase, alanine aminotransferase, alkaline phosphatase, lactate dehydrogenase (LDH), coagulation tests, cardiologic work up with electrocardiogram and echocardiogram. Every month pts underwent physical examination, blood cells count and chemistry, cardiological work up and CT-scan every three months. Responses were assessed or reviewed according to the Response Evaluation Criteria in Solid Tumors (RECIST, version 1.1). Dabrafenib was administered orally at doses of 150 mg b.i.d., under fasting conditions, either one hour before or two hours after a meal. Treatment was discontinued in cases of unacceptable toxicity, patient’s refusal or disease progression. Further, treatment beyond progression was considered in cases of clinical benefit (single site progression, manageable with local treatment, such as radiotherapy or surgery, or slow progressive disease). Adverse events were assessed on the basis of the Common Terminology Criteria of the National Cancer Institute (CTCAE, version 4.0). According to the adverse event management guidelines, Dabrafenib dose reduction was modified upon appearance of grade 3 toxicity surely related to therapy, or of complicated fever, or of toxicity considered clinically significant in the opinion of the treating physician. The dose was decreased by 50 mg b.i.d. to the minimum dose of 50 mg b.i.d. All procedures were followed in accordance with the Helsinki Declaration of 1975 (revised in 1983).

## Statistical methods

We began retrieving information on 18th April 2012, when the first patient started therapy. In October 2013, the expanded access program with the combination of Dabrafenib and Trametinib was activated for new pts and extended to all pts who were being successfully treated with Dabrafenib, without experiencing progressive disease at the time. For this reason, analysis was stopped on 31st October 2013, before pts started the combination therapy and four months after the inclusion of the last patient. All pts were evaluable for response and toxicity.

Overall response rate (ORR) is the rate of pts considered in complete remission (CR), partial remission (PR) or stable disease (SD), on the basis of the best response recorded.

Median and ranges are presented for continuous variables, and frequencies for categorical variables. OS is defined as the time interval from the date of the first Dabrafenib assumption until death or last date of follow-up, in case of censored observations. PFS was defined as the time interval from the first Dabrafenib assumption to the detection of progression or to death from any cause, whichever occurred first. Last visit date was used in case of censored observations. Survival curves were estimated using the Kaplan-Meier method. A Cox proportional hazards model was used to identify independent predictors of PFS, with adjustment for relevant confounders.

All statistical tests were two-sided, and P < 0.05 was considered statistically significant. The statistical analyses were performed by means of the Statistical Analysis System Version 9.2 (SAS Institute, Cary, NC).

## Results

The characteristics of the 40 pts included in the analysis are shown in Table 1. Median age was 55 yrs (range: 25–81 yrs) and PS was 0–1 in 67% of cases. All pts had metastatic disease at the time of treatment: four (10%) pts had one metastatic site, 13 (32%) had two metastatic sites and 23 (58%) had three or more metastatic sites. Most frequent metastatic sites were lymph nodes (62%) and lungs (57%), while CNS metastases were detected in 14 (35%) pts. All pts with CNS metastases had received whole brain (10 pts) or cyber-knife (two pts) radiotherapy before starting therapy, except for two: in one patient the lesion was surgically removed, while the other, with a small and asymptomatic lesion, was directly started on Dabrafenib. The BRAF mutation was detected on metastatic sites in 35 pts and on the primary melanoma in 5 cases. Before therapy, LDH levels were normal in 18 pts and elevated in 22 pts. Dabrafenib was the first line therapy for 29 pts and the second line for 11 pts. Previous treatments were chemotherapy (DTIC; Temozolomide; Cis-platinum-Vindesine-DTIC combination; albumin-bound Paclitaxel) in four, immunotherapy (Ipilimumab; Rec-PRAME [15]) in five, combinations of chemo-immuno or target-therapies (Ipilimumab + Fotemustine; DTIC + E7080 [16]) in two pts. All pts received at least 95% of expected dose of Dabrafenib, for a median time of 6 months (range: 1–16 months).

Toxicities were usually mild (Table 2). Cutaneous toxicities and fever were the main types of toxicity, recorded in 75% and 62% of pts, respectively. PPH/PPE and cutaneous keratosis were the most common adverse events, sometimes limiting daily activity levels, but manageable mainly with curettage and topic keratolitics (Urea 30–50). Grade 1–2 fatigue and arthralgia, recorded in 47% and 32% of pts, respectively, and were managed with oral administration of NADs or low doses of steroids. Conversely, transient fever was managed with paracetamol and/or ibuprofen and/or discontinuation for one or two days of drug administration.

Two pts required temporary discontinuation and dose reduction to 100 mg b.i.d because of recurrent fever ≥ 38°C, complicated by hypotension and chills.

Grade 3–4 toxic effects were infrequent and were recorded in 4 pts (10%). The first patient developed bilateral optic neuritis. Despite the discontinuation of Dabrafenib, visual impairment persisted. Furthermore, all examinations failed to demonstrate Horton arteritis or other causes related to therapy. Considering that the patient was experiencing disease regression, Dabrafenib was restarted after two weeks without dose adjustment. The second patient developed acute appendicitis with peritonitis, requiring surgery. Dabrafenib was interrupted until complete recovery and restarted after three weeks without dose adjustment. The third patient developed febrile neutropenia, requiring one week of therapy interruption and dose reduction to 100 mg b.i.d. The fourth patient developed an asymptomatic pulmonary thrombo-embolysm (PTE) that did not require any therapy modification.

A total of 17 skin lesions in six pts were surgically removed: one papilloma, two keratinous cysts, two warts, three seborrheic keratoses, two keratoachantomas, one pyogenic granuloma, one chronic dermatitis, one capillary angioma and four basal cell carcinomas (all pre-existing).

**Figure 1.**
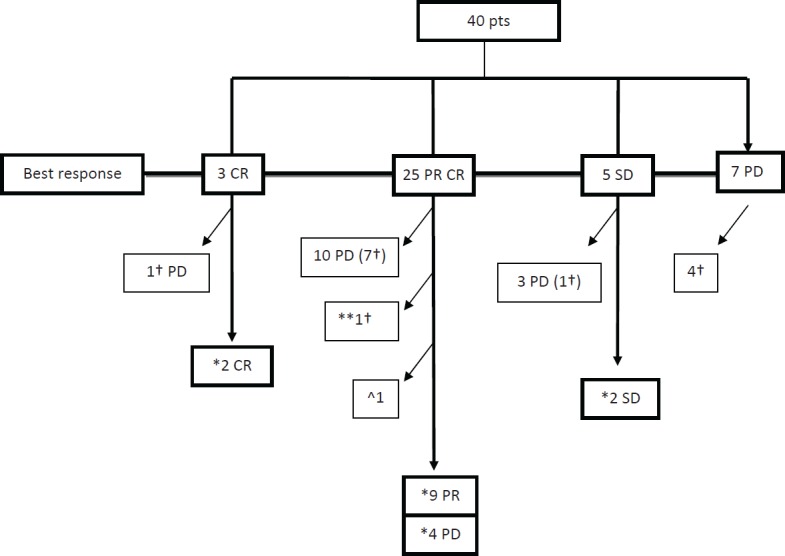
Clinical outcome. CR: complete remission, PR: partial remission, SD: stable disease, PD: progressive disease; ^†^pts dead due to melanoma; ^*^Dabrafenib ongoing therapy; ^**^pt dead due to other causes than melanoma; ^^^discontinuation due to concomitant gastric adenocarcinoma progression.

**Figure 2.**
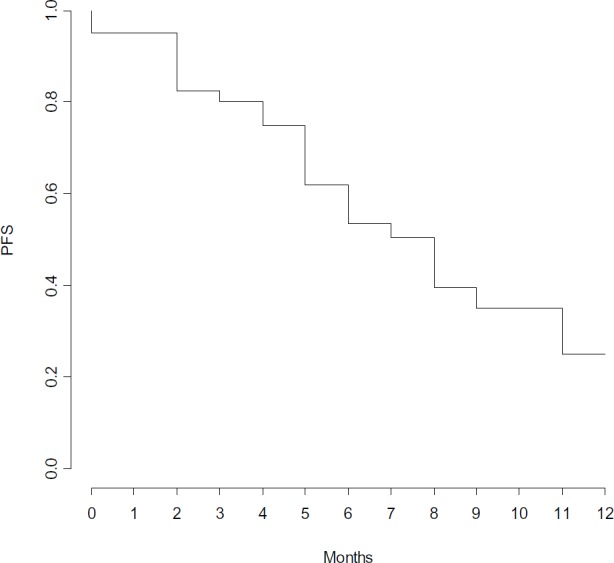
Progression free survival.

**Figure 3.**
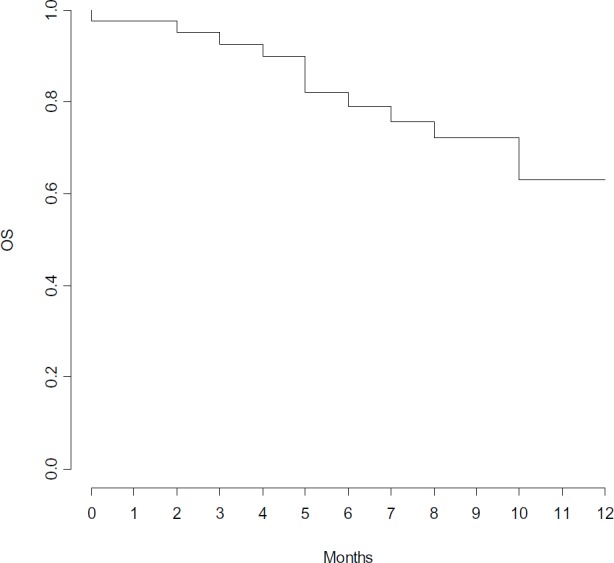
Overall survival.

Clinical outcome is reported in Figure 1. ORR was 82%, with three (7%) CR, 25 (62%) PR and five (13%) SD. Seven (18%) pts had PD within the first three months of therapy. With a median follow-up of 8.5 months, median PFS and OS were 7 and 17 months, respectively (Figure 2 and 3).

At the time of this analysis, 17 pts were still in therapy with Dabrafenib, with a median duration of treatment of 8 months (range: 4–17 months). Four out of 17 pts continued therapy with benefit, in spite of the slowly progressive disease after partial response, for a median of 8 months (range 3–13 months): one patient had liver progression with subsequent stabilization, without any further treatment; two pts had subcutaneous progression and were treated with radiotherapy in one case and surgery in the other; one patient had CNS metastases and was treated with cyber knife radiotherapy. Trametinib was added in 14 out of 17 pts, after a median of 10 months (range: 4–18 months) of Dabrafenib: one patient was in CR, 3 pts were in SD and 10 in PR. The other three pts went on with Dabrafenib alone for concomitant disease (visual impairment) or for recent PD.

Twenty-one pts stopped Dabrafenib due to disease progression. Six pts received Ipilimumab, seven chemotherapy and eight no further treatment due to rapid PD. One patient discontinued therapy because of a progressive concomitant gastric adenocarcinoma, diagnosed two months after starting Dabrafenib. A subtotal gastrectomy was performed, and the disease resulted RAF and RAS wild-type. The patient was considered not suitable for adjuvant chemotherapy and Dabrafenib was restarted. Treatment was definitely stopped after adenocarcinoma relapse.

Thirteen pts died because of melanoma, while one patient died because of surgical complications after femur fracture.

**Figure 4.**
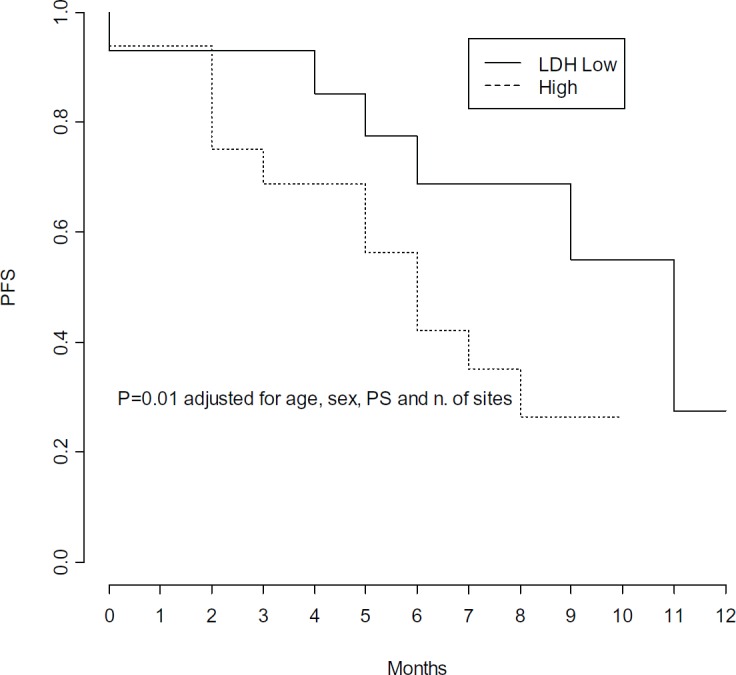
Progression free survival by level of LDH pre-treatment.

**Figure 5.**
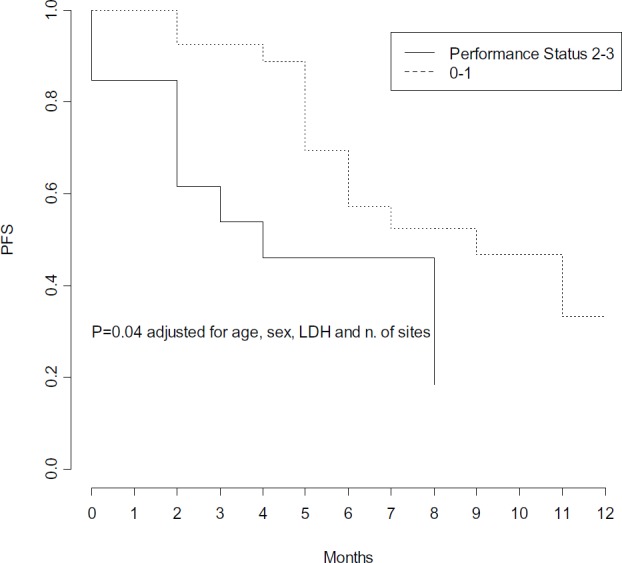
Progression free survival by performance status.

**Figure 6.**
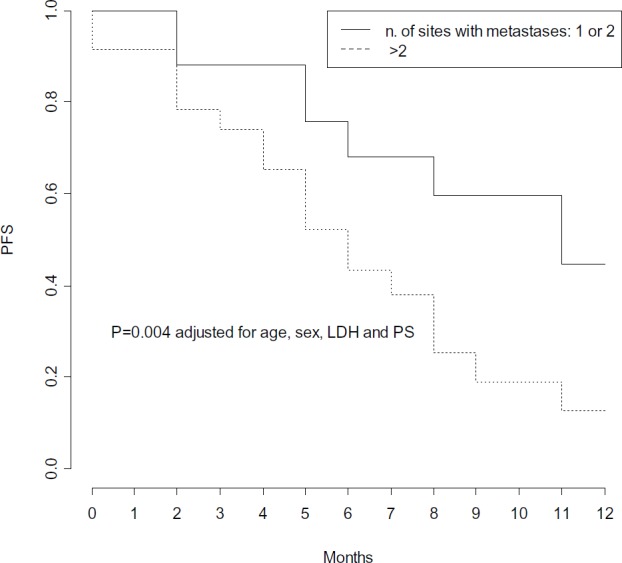
Progression Free Survival by number of sites involved with metastases.

In multivariate Cox models, few factors were found independently associated with PFS: number of metastatic sites, LDH levels and PS, adjusted for age and gender (Table 3). High pre-treatment LDH levels were independently associated with a significant 4-fold higher risk of progression (p = 0.01) (Figure 4), while PS lower than 2 was independently associated with a 68% decreased risk of progression (p = 0.04) (Figure 5), and a statistically significant 77% decreased risk of progression was found in the presence of no more than 2 metastatic sites (p = 0.004) (Figure 6).

## Discussion

The outcome of this retrospective analysis confirms the findings of clinical studies on the effect of Dabrafenib therapy in ‘real life experience’ in pts with MM, as already reported in randomized [7, 8] or observational trials [17]. Four pts (10%) developed G3-4 adverse events, not all definitely related to Dabrafenib assumption, such as peritonitis and bilateral optical neuritis, which caused temporary therapy interruptions or dose reductions. The incidence of neoplastic skin lesions and photosensitivity were infrequent and their rate was similar to that already reported in the literature [7]. The other relevant toxic effects such as transient G1-2 fever, fatigue and arthralgia, occurred more frequently than reported, probably due to the small simple size. However, they did not affect compliance to treatment and were easily manageable.

One pt developed a gastric adenocarcinoma, evident after two months of therapy. There are many concerns about the use of BRAF inhibitors in the presence of epithelial cancers. Gastric cancers could harbor RAS mutation in 8–25% of cases [18] and its presence could induce a paradoxical MAPK pathway activation during BRAF inhibitors therapy. In this case we decided to continue Dabrafenib, preferring to treat the metastatic melanoma more than shifting to adjuvant chemotherapy because of RAS and BRAF-WT gastric cancer.

Considering that this is a small series of unselected melanoma pts and some of them added Trametinib during Dabrafenib, survival data might be interpreted with caution. Anyhow, clinical results also seemed to be comparable to reported data [7, 17]. The major benefit from treatment was more evident in pts in good general conditions, with low tumor burden and normal LDH. These parameters are well known poor prognostic factors, and increased LDH was found to be associated with a poorer outcome in pts receiving Dabrafenib alone [11]. Elevated LDH constitutes an unfavoureable prognostic factor also in pts treated with Dabrafenib and Trametinib combination instead of Vemurafenib, with a modest advantage in terms of PFS in favour of combination [19]. The majority (5/7) of pts who progressed within the first three months of therapy were those who initially presented with a high tumor burden, with at least two metastatic sites (range 2–5), high LDH level and PS ≥2.

Although the ORR was 82%, only 7% of pts experienced CR as best response, which suggests a possible correlation with the relatively short duration of disease control (7 months).

Several studies demonstrated an advantage in terms of OS for patients treated with BRAF inhibitors in spite of slow and/or limited progression [20, 21]. In our analysis, four pts with an asymptomatic single lesion progression (cutaneous, liver, CNS) received Dabrafenib beyond progression. All these pts were immunotherapy naïve, and the issue about when and whether it is better to modify treatment strategy in ‘real life experience’ is still unclear. In our series target therapy gave a further meaningful disease control, without recurring to other treatment options, for a median of eight months and about for one year in two cases, suggesting that in this setting, a single pt decision is mandatory.

MEK inhibitors were combined with BRAF inhibitors in order to improve their efficacy. Three randomized clinical trials comparing BRAF inhibitor monotherapy with BRAF/MEK inhibitors association were recently published, demonstrating a better outcome for combinations. In the COMBI-D trial [11], the Dabrafenib/Trametinib combination versus Dabrafenib alone improved PFS (9.3 versus 8.8 months; HR = 0.75; P = 0.03) and ORR (67% versus 51%, respectively; P = 0.002). In the Co-BRIM trial [12], the Vemurafenib/Cobimetinib combination versus Vemurafenib alone improved PFS (9.9 versus 6.2 months; HR = 0.51; P < 0.001) and ORR (68% versus 45%; P < 0.001). In the COMBI-V trial [13], the Dabrafenib/Trametinib combination versus Vemurafenib alone improved PFS (11.4 versus 7.3 months; HR = 0.56; P < 0.001) and ORR (64% versus 51%, respectively; P < 0.001).

The Dabrafenib/Trametinib combination seems also to modify the toxicity profile of Dabrafenib alone, by reducing the incidence of cSCC and other cutaneous proliferative disorders [11, 14]. Thus, with the activation of the Compassionate Program for Dabrafenib and Trametinib combination, Trametinib was added to therapy in 14 ongoing and responding pts.

Target therapies appear to be still inadequate for long term disease control, although combination Dabrafenib and Trametinib could achieve 3-years survival rate of 32% [22].

Several observations focused attention on the immunomodulatory effect of BRAF/MEK inhibitors, that induce an increase of melanoma antigens expression and of CD8+ lymphocyte intratumoral infiltration, and a reduction of the release of several immunosuppresive cytokines [23]. Speculating that these mechanisms could be crucial for long term disease control, the combination of target therapies with checkpoint inhibitors like anti-CTLA4 [24], anti- PD-1 [25] and PD-L1 [26] antibodies, could further increase the immunomodulatory effect and represents an intriguing research area.

In clinical practice, target therapy constitutes the first line of treatment for symptomatic pts in reason of their faster and effective responses, while checkpoint inhibitors are more often used in case of indolent disease.

However, the best strategy for BRAF V600 mutated pts is still unknown and under investigation. Several ongoing randomized trials are focused on the identification of the best upfront therapy between Dabrafenib/Trametinib and Ipilimumab/Nivolumab (NCT02224781). On the other hand, possible strategies could provide an upfront immunotherapy/target therapy combination with Dabrafenib/Trametinib/ Pembrolizumab and Pembrolizumab (NCT02130466), or a sandwich modality, shifting to Ipilimumab/Nivolumab combination after an induction phase with BRAF/MEK inhibitors (NCT02631447). Therefore, antibodies, molecular agents and vaccines widened other perspectives in the management of metastatic melanoma, widening the possible therapeutic options.

## Conclusion

The combination of BRAF/MEK inhibitors like Dabrafenib and Trametinib might be considered one of the standard clinical options in metastatic BRAF V600 mutated pts.

The combined regimen has shown to be both more effective and less toxic than BRAF inhibitors alone. However, the combination is more expensive and not yet available in all countries. In such a scenario, Dabrafenib as monotherapy should be preferred in those patients who are more likely to develop skin side effects, such as photosensivity, which may compromise patients’ quality of life and their compliance to therapy, especially for those living in very sunny areas. Further, data from ongoing randomized trials will clarify how to better combine the available drugs, in order to achieve the best clinical benefit.

## Declaration of interests

Emilia Cocorocchio: Consultant for Bristol-Myers Squibb, GlaxoSmithKline. Massimo Barberis: Consultant for GlaxoSmithKline. Pier Francesco Ferrucci: Consultant and Advisory Role for Roche, Bristol-Myers Squibb, GlaxoSmithKline. For the other authors there are no conflicts of interest to disclose.

## Figures and Tables

**Table 1.** Characteristics of patients included in the analysis.

Pts characteristics		
**# Pts**		40 (100%)
**Age**	Median Range	55 yrs 25–81
**Performance Status**	0–1 2 3	27 (67%) 5 (13%) 8 (20%)
**Gender**	Males	28 (70%)
	Females	12 (30%)
**Stage**	M1a M1b M1c	2 (5%) 1 (3%) 37 (92%)
**# Metastatic sites**	1 2 ≥3	4 (10%) 13 (32%) 23 (58%)
**Metastatic sites**	Lung	23 (57%)
	Liver	12 (30%)
	Spleen	3 (7%)
	Lymph-nodes	25 (62%)
	Skin/subcutaneous	16 (40%)
	Central Nervous System	14 (35%)
	Other	16 (40%)
**# previous therapy lines**	0 1	29 (72%) 11 (33%)
**LDH pre-treatment**	Normal	18 (45%)
	Elevated	22 (55%)

**Table 2.** Frequencies of toxicity (CTCAE criteria).

Hematological toxicity	G 1–2 (%)	G 3–4 (%)
Leucopenia	2 (5%)	0
Neutropenia	2 (5%)	1^*^ (2%)
Anemia	13 (32%)	0
Thrombocytopenia	1 (2%)	0
**Non haematological toxicity**		
Fever	25 (62%)	0
Diarrhoea	3 (7%)	0
Cutaneous Keratoses ^ψ^ PPH/PPE Alopecia Keratoachantoma Basal cell carcinoma Others ^§^	30 (75%) 26 (65%) 8 (20%) 5 (12%) 1 (2%) 2 (5%) 4 (10%)	0 – – – – – –
Artralgia	13 (32%)	0
Hypotension	3 (7%)	0
Fatigue	19 (47%)	0
Other	11^**^ (27%)	3^^^ (7%)

* Febrile neutropenia.

Ψ cutaneous keratoses, xerosis, achantoma.

§ Folliculitis, photosensitivity.

** Mucositis, transaminitis, nausea/vomiting, epigastric pain.

^ Pulmonary thromboembolism; acute appendicitis with peritonitis, bilateral optic neuritis. PPH/PPE: Palmo-plantar hyperkeratosis/erytrodysaesthesia.

**Table 3.** Results from multivariate Cox models.

		HR	Low 95%CI;	Up 95%CI;	P-values
**LDH pre**	High versus Low	4.26	1.42	12.7	0.010
**n. met.**	≤2 versus >2	0.23	0.08	0.62	0.004
**PS**	0–1 versus 2–3	0.32	0.11	0.97	0.044
**Age**	–	0.98	0.95	1.01	0.234
**Gender**	Men versus Women	2.43	0.75	7.94	0.140

n. met.: number of metastatic sites. PS: Performance Status. LDH pre: LDH pre-treatment.
